# Supplementary material for: Evidence of reduced viremia, pathogenicity and vector competence in a re‐emerging European strain of bluetongue virus serotype 8 in sheep
Source: Transbound Emerg Dis. 2019 Feb 22;66(3):1177–85. doi: 10.1111/tbed.13131 (PMC6563110; doi:10.1111/tbed.13131)
Supplement: Supplementary file 1 [file TBED-66-1177-s001.docx]

**Supplementary information: Randomisation of animals**

The sheep were randomly assigned to two groups blocked by their weight and the number of sites where specific clinical signs of BTV could be assessed (i.e. pathological changes to mucosal membranes due to the dark pigmentation of the mucosa membranes in some of the sheep)

Sheep with a weight greater than the median (range 60 to 86 Kg) were categorized as heavy.

These covariates were selected as weight was taken as a readout of overall nutritional state and body condition, while the colour of the mucosal membranes might interfere with the scoring of mild clinical signs.

The block_ra function in R randomizr package was used to assign the sheep to either challenge group.
