# Supplementary material for: Evidence of reduced viremia, pathogenicity and vector competence in a re‐emerging European strain of bluetongue virus serotype 8 in sheep
Source: Transbound Emerg Dis. 2019 Feb 22;66(3):1177–85. doi: 10.1111/tbed.13131 (PMC6563110; doi:10.1111/tbed.13131)
Supplement: Supplementary file 2 [file TBED-66-1177-s002.docx]

BTV RNA concentrations in sheep blood determined by RT-qPCR

|  | Log_10_ BTV genome copies ml^-1^ EDTA blood (inoculated with UKG2007) | | | | | | | | |  | Log_10_ BTV genome copies ml^-1^ EDTA blood (inoculated with FRA2017) | | | | | | | | |
| --- | --- | --- | --- | --- | --- | --- | --- | --- | --- | --- | --- | --- | --- | --- | --- | --- | --- | --- | --- |
| DPI | Sheep 1 | Sheep 2* | Sheep 3 | Sheep 4 | Sheep 5 | Sheep 6 | Sheep 7 | Sheep 8 | Sheep 9^1^ |  | Sheep 10 | Sheep 11^2^ | Sheep 12 | Sheep 13 | Sheep 14 | Sheep 15 | Sheep 16 | Sheep 17 | Sheep 18* |
| -1 | Undet. | Undet. | Undet. | Undet. | Undet. | Undet. | Undet. | Undet. | Undet. |  | Undet. | Undet. | Undet. | Undet. | Undet. | Undet. | Undet. | Undet. | Undet. |
| 0 | - | - | - | - | - | - | - | - | - |  | - | - | - | - | - | - | - | - | - |
| 1 | - | - | - | - | - | - | - | - | - |  | - | - | - | - | - | - | - | - | - |
| 2 | 4.62 | Undet. | 3.43 | Undet. | Undet. | 4.75 | 4.13 | Undet. | 4.74 |  | Undet. | 3.82 | 2.09 | Undet. | Undet. | Undet. | 2.68 | Undet. | Undet. |
| 3 | 6.62 | Undet. | 5.43 | 2.81 | 2.35 | 6.93 | 6.03 | 3.18 | 7.04 |  | Undet. | 5.26 | 4.31 | Undet. | Undet. | 2.13 | 3.74 | Undet. | Undet. |
| 4 | - | - | - | - | - | - | - | - | - |  | - | - | - | - | - | - | - | - | - |
| 5 | 7.47 | Undet. | 6.78 | 4.93 | 5.98 | 8.05 | 7.03 | 5.35 | 7.87 |  | 1.89 | 7.31 | 6.42 | 3.76 | 3.88 | 3.90 | 5.29 | Undet. | Undet. |
| 6 | 7.68 | Undet. | 6.53 | 5.54 | 6.99 | 8.26 | 6.71 | 6.02 | 8.04 |  | 3.81 | 7.19 | 6.72 | 5.09 | 4.74 | 4.49 | 5.65 | Undet. | Undet. |
| 7 | 7.47 | Undet. | 6.13 | 5.36 | 6.86 | 7.93 | 6.32 | 5.80 | 7.71 |  | 4.61 | 7.30 | 6.59 | 5.73 | 5.06 | 4.45 | 5.29 | Undet. | Undet. |
| 8 | 7.28 | Undet. | 5.86 | 5.09 | 6.85 | 7.77 | 6.07 | 5.45 | 7.40 |  | 4.79 | 6.84 | 6.56 | 6.04 | 5.12 | 4.65 | 5.13 | Undet. | Undet. |
| 9 | 7.27 | Undet. | 5.87 | 4.87 | 6.62 | 7.44 | 5.94 | 5.15 | 7.02 |  | 4.77 | 6.40 | 6.43 | 6.11 | 4.76 | 4.15 | 4.87 | Undet. | Undet. |
| 10 | 6.82 | Undet. | 5.38 | 4.33 | 6.33 | 6.95 | 5.47 | 4.55 | - |  | 4.24 | 5.56 | 6.17 | 5.69 | 4.49 | 3.51 | 4.58 | Undet. | Undet. |
| 11 | - | - | - | - | - | - | - | - | - |  | - | - | - | - | - | - | - | - | - |
| 12 | 6.21 | Undet. | 5.39 | 4.52 | 5.56 | 6.62 | 5.41 | 4.78 | - |  | 3.54 | 5.10 | 5.83 | 5.34 | 3.95 | 3.18 | 4.44 | Undet. | Undet. |
| 13 | - | - | - | - | - | - | - | - | - |  | - | - | - | - | - | - | - | - | - |
| 14 | 5.87 | Undet. | 5.17 | 4.00 | 5.27 | 6.43 | 5.14 | 4.57 | - |  | 3.48 | 5.00 | 5.43 | 4.71 | 3.63 | Undet. | 3.97 | Undet. | Undet. |
| 15 | - | - | - | - | - | - | - | - | - |  | - | - | - | - | - | - | - | - | - |
| 16 | 5.65 | Undet. | 5.00 | 3.41 | 5.26 | 6.27 | 5.07 | 4.27 | - |  | 2.98 | 5.15 | 5.29 | 4.47 | 3.43 | Undet. | 2.97 | Undet. | Undet. |
| 17 | - | - | - | - | - | - | - | - | - |  | - | - | - | - | - | - | - | - | - |
| 18 | - | - | - | - | - | - | - | - | - |  | - | - | - | - | - | - | - | - | - |
| 19 | 5.69 | Undet. | 5.12 | 2.71 | 5.20 | 6.35 | 5.18 | 4.14 | - |  | 2.58 | - | 5.15 | 4.25 | 3.32 | Undet. | 2.29 | Undet. | Undet. |
| 20 | - | - | - | - | - | - | - | - | - |  | - | - | - | - | - | - | - | - | - |
| 21 | 5.70 | Undet. | 5.12 | 1.85 | 5.13 | 6.38 | 5.13 | 4.08 | - |  | Undet. | - | 4.97 | 4.02 | 2.63 | Undet. | Undet. | Undet. | Undet. |

*Control sheep, ^1^Sheep was euthanized at 9 dpi, ^2^Sheep was euthanized at 16 dpi, - Sheep was not sampled, Undet. Undetected by RT-qPCR
